# Atmospheric pressure field desorption-trapped ion mobility-mass spectrometry coupling

**DOI:** 10.1007/s00216-024-05282-0

**Published:** 2024-04-08

**Authors:** Jürgen H. Gross

**Affiliations:** Institute of Organic Chemistry, Heidelberg University, Im Neuenheimer Feld 270, 69120 Heidelberg, Germany

**Keywords:** Atmospheric pressure field desorption (APFD), Trapped ion mobility spectrometry (TIMS), Field desorption (FD), Field ionization (FI), Emitter heating current (EHC), Activated field emitter, Ambient desorption/ionization (ADI), Trapped ion mobility-quadrupole-time-of-flight (TIMS-Q-TOF)

## Abstract

While field ionization (FI) and field desorption (FD) are established soft vacuum ionization methods in mass spectrometry (MS), the technique of atmospheric pressure field desorption (APFD) has only recently been added to the repertoire. Similar to FI and FD, APFD can yield both positive even-electron ions of highly polar or ionic compounds and positive molecular ions, M^+•^, e.g., of polycyclic aromatic compounds. Thus, a dedicated APFD source assembly has been constructed and demonstrated to allow for robust APFD operation. This device also enabled observation of the emitter during operation and allowed for resistive emitter heating, thereby speeding up the desorption of the analytes and expanding the range of analytes accessible to APFD. While initial work was done using a Fourier transform-ion cyclotron resonance (FT-ICR) mass spectrometer, the new APFD source offered the flexibility to also be used on a trapped ion mobility-quadrupole-time-of-flight (TIMS-Q-TOF) instrument, and thus, it would be possible to be mounted to any Bruker mass spectrometer featuring an atmospheric pressure (AP) interface. Operating an APFD source at a TIMS-Q-TOF instrument called for the exploration of the combined use of APFD and TIMS. Here, operation, basic properties, and capabilities of this new atmospheric pressure field desorption-trapped ion mobility-mass spectrometry (APFD-TIMS-MS) coupling are described. APFD-TIMS-MS is employed for the separation of individual components of oligomers and for the accurate determination of their collision cross section (CCS). This work describes the application of APFD-TIMS-MS on poly(ethylene glycol) forming [M + Na]^+^ ions by cationization and on an amine-terminated poly(propylene glycol) yielding [M + H]^+^ ions. Some compounds forming molecular ions, M^+•^, by field ionization such as [60]fullerene and a mixture of four polycyclic aromatic hydrocarbons (PAHs) are examined. In APFD-TIMS-MS, the limits of detection (LODs) of fluoranthene and benzo[a]pyrene M^+•^ ions are determined as ≈100 pg and <1 pg, respectively. Finally, [60]fullerene is analyzed by negative-ion APFD-TIMS-MS where it yields a molecular anion, M^−•^.

**Graphical abstract:**

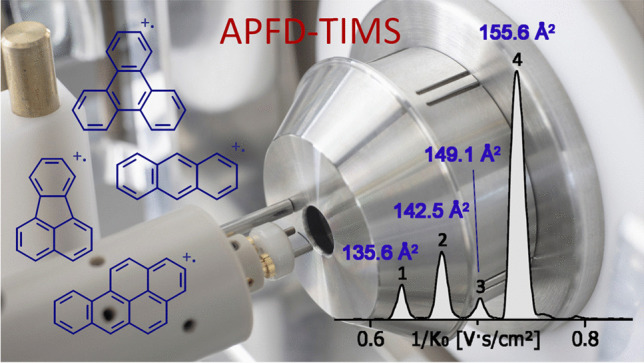

**Supplementary Information:**

The online version contains supplementary material available at 10.1007/s00216-024-05282-0.

## Introduction

Both field ionization (FI) and field desorption (FD) are known for decades as very soft vacuum ionization methods in mass spectrometry (MS) [1–4]. The reason for running the ionization process in high vacuum is that the mechanism of field ionization relies on very strong electric fields in the order of 1–2 V Å^−1^ that typically necessitate high voltage of about 10 kV to be applied across a 2-mm gap between the field emitter and a counter electrode to occur [1, 5]. In practice, the extreme electric field strengths needed to effectively generate M^+•^ ions by the FI process are achieved by using so-called activated field emitters [6–8]. Unfortunately, electric discharges tend to result in the destruction of the emitter, and thus, are limiting the voltages that can be applied to achieve the highest possible electric field strengths.

The FI process yields intact positive molecular ions, M^+•^, while the FD process transfers preformed ions such as protonated molecules, [M + H]^+^, and/or alkali ion adducts, [M + alkali]^+^ into the gas phase [1–4]. FD of preformed ions requires field strengths that are about two orders of magnitude lower than those needed for FI [9–13]. Moving from high vacuum into other pressure regimes has therefore been considered futile for the most part. Thus, only a handful of studies were conducted using either super-atmospheric pressure [13] or were presenting isolated cases of FD at atmospheric conditions [14, 15].

Inspired by those rare exceptions and by various publications in the field of ambient desorption/ionization (ADI) [16–18], in particular those where strong electric fields played a role for analyte ion desorption to occur [19–24], a systematic exploration of the technique of atmospheric pressure field desorption (APFD) has been conducted. Surprisingly, activated field emitters of the very same type as used in vacuum FI or FD could be shown to deliver sufficiently strong electric fields to allow for FI and FD at atmospheric pressure. To achieve this, voltages in the order of 4.5–5.5 kV were generally sufficient [25]. Like in FI and FD, positive even-electron ions of highly polar or ionic compounds and even positive molecular ions, M^+•^, e.g., of polycyclic aromatic compounds, could be generated in APFD [26]. Furthermore, APFD could be employed in negative-ion mode to yield negative even-electron ions of highly polar or ionic compounds like surfactants in commercial detergents [27].

It became clear that the further advancement of APFD would benefit from a more robust and reproducible means of positioning and aligning the emitter. Encouraged by the results obtained so far [25–27], a dedicated APFD source assembly has been constructed and demonstrated to allow for robust APFD operation. This device also enabled observation of the emitter during operation and allowed for resistive emitter heating as in traditional FD [28–30], thereby expanding the range of analytes accessible to APFD [31].

While initial APFD work was done using a Fourier transform-ion cyclotron resonance (FT-ICR) mass spectrometer, the new APFD source offered the flexibility to also be used on a trapped ion mobility-quadrupole-time-of-flight (TIMS-Q-TOF) instrument, and thus, it would be ready to be mounted to any current Bruker mass spectrometer featuring an atmospheric pressure (AP) interface [31].

The coupling of ion mobility separation and mass spectrometry became generally known more than two decades ago [32–37] and by now is well-established in various technical implementations [38]. Among these, the instrument used here relies on trapped ion mobility separation (TIMS) [39–42].

Operating an APFD source on a TIMS-Q-TOF instrument called for the exploration of the combined use of APFD and TIMS. Here, operation, basic properties, and capabilities of this new combination are described. APFD-TIMS-Q-TOF–MS is employed for both ion mobility separation and determination of collision cross sections (CCS) across a range of compound classes. The present study describes the application of the APFD-TIMS combination on polar oligomers forming even-electron ions, i.e., either [M + H]^+^ ions by protonation or [M + Na]^+^ ions by cationization, and on [60]fullerene and a mixture of four polycyclic aromatic hydrocarbons (PAHs) forming molecular ions, M^+•^, by field ionization. An example of negative-ion APFD-TIMS is presented by the analysis of [60]fullerene that yields M^−•^ ions.

## Experimental

### Mass spectrometer

A trapped ion mobility-quadrupole-time-of-flight (TIMS-Q-TOF) instrument (timsTOFflex, Bruker Daltonics, Bremen, Germany) was used. The instrument was equipped with an ESI-to-MALDI switchable dual source. According to the manufacturer, the AP interface of all current Bruker instruments is identical to the above, and thus, the APFD source is fully compatible among these. The mass spectrometer was controlled by the Bruker timsControl software (V 2.0) and data analysis was performed using the Bruker DataAnalysis software (V 6.0).

External mass calibrations were established in ESI mode by using Agilent Tune Mix (G1969-85,000) for the *m*/*z* 100–2500 range [43, 44]. Mass accuracy was in the order of 3 ppm.

### APFD source

**Fig. 1:**
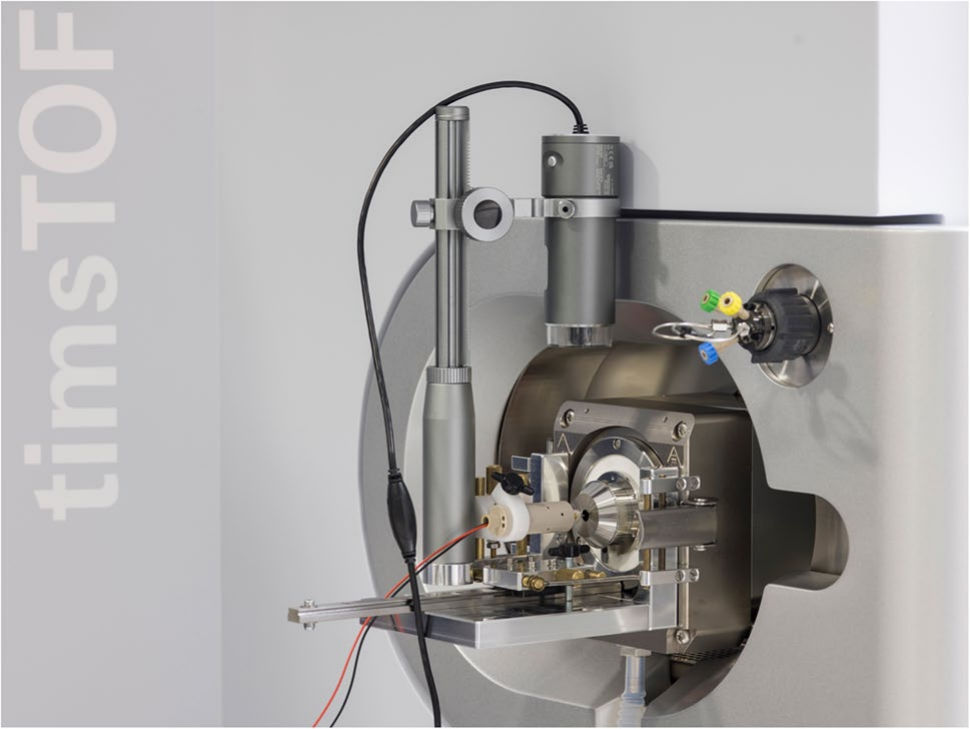
Photograph of the APFD source mounted to the AP interface of the timsTOFflex instrument. The probe holder stage carrying the emitter is in the position for APFD operation. The fine red and black wires are connected to the DC power supply for providing the EHC

The APFD source design has recently been published in detail [31] and shall only briefly be described here. For APFD operation, the instrument manufacturer’s ESI sprayer is swapped for the APFD assembly that is based on an aluminum frame which fits the hinges and source clamp of the AP interface of the timsTOFflex mass spectrometer. The frame supports a rail allowing an *x*,*y*-adjustable mounting stage that carries the APFD probe to slide along the *z*-axis (Fig. 1). The emitter can thus be pushed forward to the spray shield electrode for APFD operation or be pulled back for emitter loading or exchange. The APFD probe comprises emitter sockets with electric feed-through for the emitter heating current (EHC) and a pin to adjust the distance of the emitter to the spray shield (2.0 mm) that is acting as the counter electrode. The mounting stage carries a USB microscope (DST-1028, Bresser, Rhede, Germany) enabling emitter observation from top during the entire operation. The EHC is supplied by a regulated power supply (MPD-6015 DC, Manson, Hong Kong, China). A more detailed look at the mounting stage in retracted and operational position is provided as Figs. S1–S2 in the Supplementary Material.

### APFD operation

The emitter was positioned horizontally in the center of the aperture of the spray shield electrode of the Bruker AP interface where the spray shield served as the counter electrode for the emitter. Underneath, a rounded metal cap is mounted on the glass transfer capillary. While the emitter always remained at ground potential, high voltage was only applied to the counter electrode provided by the API interface and was set using the API source controls. In positive-ion APFD, both voltages were negative to attract positive ions whereas in negative-ion APFD they were positive to attract anions. In practice, the spray shield voltages were in the range of ± 3.5 to ± 5.0 kV while the cap underneath the spray shield was set to 100–500 V higher voltage. The drying gas at the spray shield was set to 2.0–4.0 l min^−1^ at 100–250 °C. Other instrument settings were the same as in ESI operation. Caution: This work uses a prototype source where high voltage is applied to the spray shield without cover. Basic care provided, the user is nonetheless safe from getting into contact with high voltage during operation as all other parts are at ground potential.

A 10-µl syringe was used to manually deliver the sample solutions the emitter. Analytes were prepared as solutions of concentrations of 0.5–2 mg ml^−1^. After the solvents had completely been evaporated, the emitter was moved forward to the position for operation. Between the runs, the emitter was either baked using the EHC at about 160 mA or occasionally rinsed with solvent to wash off excessive analyte. The activated field emitters were of the standard type commercially available for the JEOL AccuTOF series of instruments [45, 46] and were based on 13-µm tungsten wires (Linden CMS, Weyhe, Germany). Typically, an emitter could be used for tens of acquisitions.

### Acquisition of APFD-TIMS spectra

The TIMS calibration was performed with the electrospray source as the calibration routine of the timsTOFflex instrument needs a continuous ion current across the entire calibration range. As emitter heating in APFD goes along with some temporal fractionation of the sample, APFD is not suitable for TIMS calibration. The TIMS calibration was performed following the standard procedure of the instrument that is based on Agilent ESI Tune Mix (G1969-85,000). For TIMS calibration of the high-mobility range, an admixture of Agilent APCI Tune Mix (G1969-85,010) served to enhance the reference peak at *m*/*z* 322. The manufacturer’s reference file that provides 1/*K*_0_ values for positive and negative ions of these molecules was used to calibrate the TIMS unit [47, 48]. TIMS parameters were adjusted as to achieve a balanced mix of 1/*K*_0_ range, resolving power, and sensitivity. Typical settings for PEG 300 and [60]fullerene were ion accumulation 25–150 ms, ramp time 300–400 ms, 1/*K*_0_ range 0.60–1.40, and TIMS inlet pressure 2.7 mbar. Once the TIMS calibration was completed, the ESI source was swapped for the APFD source, which could be accomplished within 2–3 min. Then, the APFD source could immediately be operated without the need for extra tuning. The ease of swapping from ESI to APFD and back allowed for recalibration of the TIMS unit whenever a different 1/*K*_0_ range or ramp time was going to be used. APFD-TIMS mass spectra were either manually extracted from the total ion mobilogram as required for the specific sample or obtained by using the function “Find Components–Mobilogram” using the default settings provided in DataAnalysis 6.0.

### Samples

A set of samples previously explored to establish the basics of APFD mode was reused here as a control of general operation and to check for reproducibility, and thus, details on most samples as well as reference APFD without TIMS were already communicated [25–27]. Solvents of LC–MS grade and polyethylene glycol 300 were obtained from Merck KGaA (Darmstadt, Germany). A sample of triphenylene was available within the author’s institution. The analytes are compiled in Table 1.

**Table 1:** Compounds analyzed by APFD-MS in the order of appearance

Compound name	Relevant ionic formulas	Calculated *m*/*z* value(s)
Poly(ethylene glycol) 300 (PEG 300)	[HO(C_2_H_4_O)_n_H + Na]^+^	305.1557, 349.1828, 393.2093, 437.2352, 481.2609, 525.2872, 569.3136, 613.3398
[60]Fullerene	[C_60_]^+•^, [C_60_]^−•^	719.9995 720.0005
Amino-terminated poly(propylene glycol) (Jeffamine M-2005)	[CH_3_O-(C_2_H_4_O)_n_(C_3_H_6_O)_m_-NH_3_]^+^	466.3738, 524.4157, 582.4576, 640.4994, 698.5413, 756.5832, 814.6250, 872.6669, …
Anthracene	[C_14_H_10_]^+•^	178.0777
Fluoranthene	[C_16_H_10_]^+•^	202.0777
Triphenylene	[C_18_H_12_]^+•^	228.0934
Benzo[a]pyrene	[C_20_H_12_]^+•^	252.0934

## Results and discussion

### Poly(ethylene glycol) sodium adduct ions

**Fig. 2:**
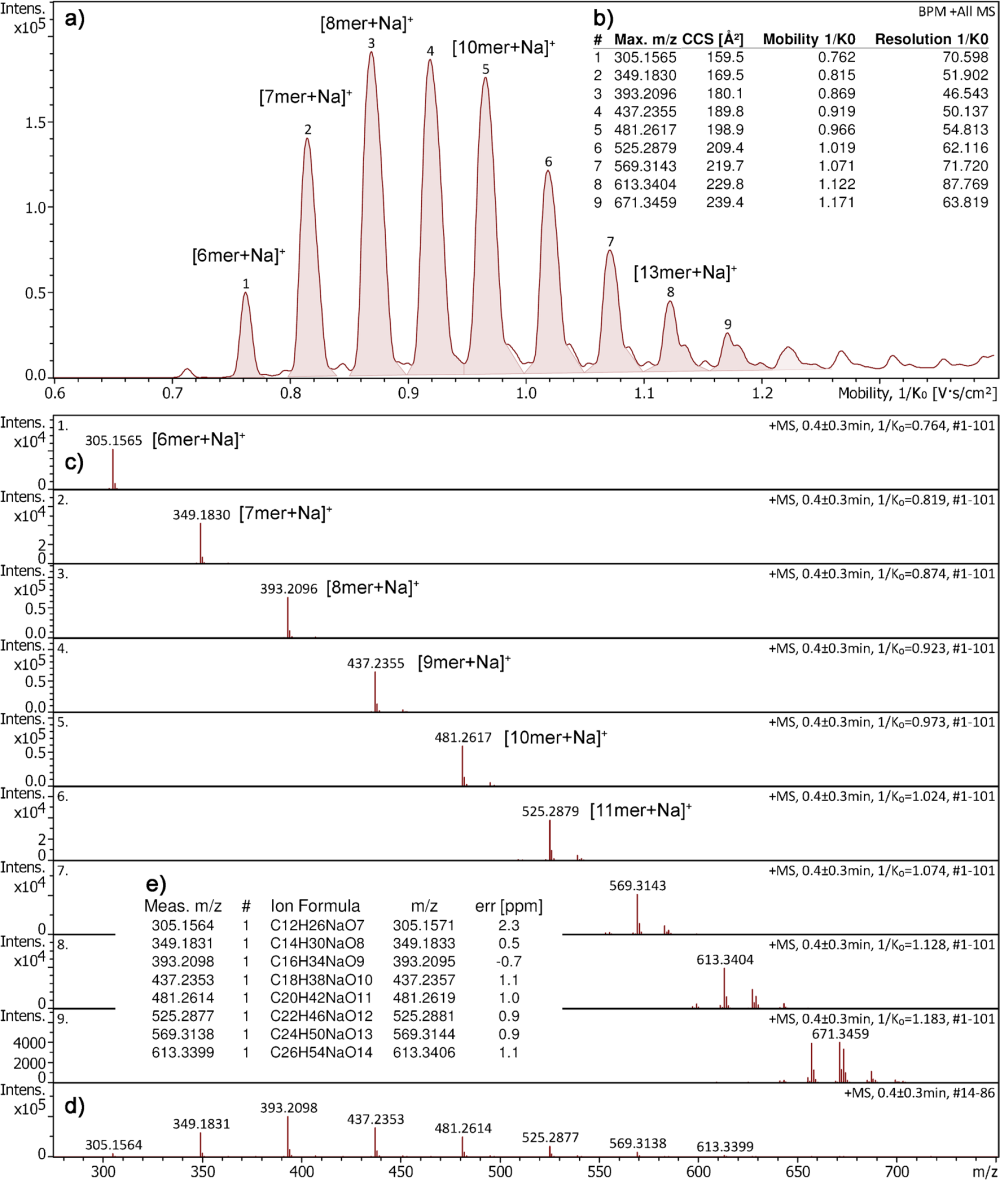
Positive-ion APFD-TIMS analysis of PEG 300. **a** Base peak ion mobilogram (BPC) with compound numbers assigned to individual peaks. **b** List of mobilogram peaks with corresponding CCS, 1/*K*_0_, and TIMS resolution data. **c** Compound spectra corresponding to the individual [*n*-mer + Na]^+^ species. **d** Mass spectrum as obtained by summing the entire range (as if there was no mobility separation) along with **e** formula assignments based on accurate mass data

As accurate collision cross sections (CCS) of various cationized individual monomers of pol(ethylene glycol) (PEG) have been published [49], PEG of average molecular weight of 300 u (PEG 300) was taken as a first test of APFD-TIMS operation in positive-ion mode. About 1 µg of PEG 300 was transferred onto the emitter by attaching a drop of about 1 µl of a solution at 1 mg ml^−1^ in methanol to it. After evaporation of the solvent, the emitter was positioned at the spray shield at − 4300 V and the acquisition was started with the TIMS in operation. During that period, the EHC was manually ramped up to 0.12 A causing the entire acquisition to take about 35 s. Meanwhile the TIMS analyzer was operated with an ion accumulation time of 100 ms, a ramp time of 400 ms across a 1/*K*_0_ range of 0.60–1.40, and an inlet pressure of 2.7 mbar. With the default settings, the compound finder algorithm assigned a total of nine compounds to the well-separated peaks of the mobilogram, corresponding to the individual monomeric PEG ions (Fig. 2). The mass spectra revealed that PEG exclusively had formed [M + Na]^+^ ions, which became also obvious from the accurate mass data. The oligomers had not only been separated by their ion mobilities; moreover, the CCS values obtained were also in very good agreement with the published ones, e.g., *m*/*z* 305.1565 with exp. 159.5 Å^2^ (161 Å^2^ in ref. [49]), *m*/*z* 349.1830 with exp. 169.5 Å^2^ (168 Å^2^), *m*/*z* 393.2026 with exp. 180.1 Å^2^ (183 Å^2^). A full evaluation of the CCS values based on a set of three sequential acquisitions is provided in the Supplementary Material (Fig. S3). It reveals that an average CCS accuracy of about 1% has been obtained without taking special care to achieve utmost accuracy, i.e., by using quite routine TIMS settings.

### [60]Fullerene

**Fig. 3:**
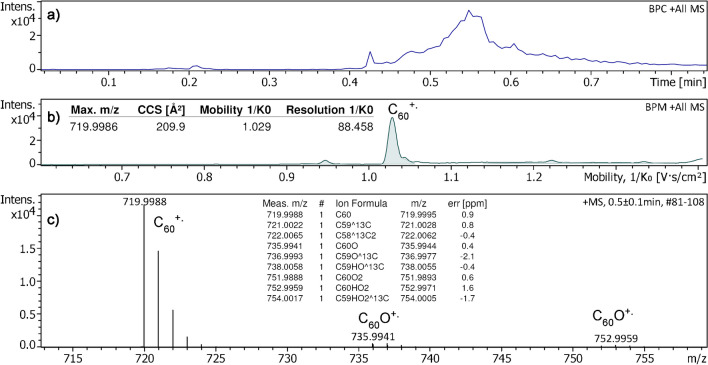
Positive-ion APFD-TIMS analysis of [60]fullerene. **a** Base peak chromatogram where the onset of [C_60_]^+•^ ion formation was when the EHC reached 0.16 A at 0.42 min. **b** Base peak mobilogram (setting B) showing the fullerene signal with corresponding CCS, 1/*K*_0_, and TIMS resolution data. **c** Mass spectrum as obtained by summing the desorption across the range of highest intensity. The insert shows the formula assignments to monoisotopic and the first two carbon isotopic ions of C_60_, C_60_O, and C_60_O_2_ based on accurate mass data. For this analysis, the spray shield was set to –4800 V and the desolvation gas to 250 °C

[60]Fullerene presented another interesting molecule to explore APFD-TIMS. On the one hand, the very low volatility of C_60_ would call for very high emitter currents that might be hard to realize at atmospheric pressure without burning off the emitter, while on the other hand, the CCS value for [C_60_]^+•^ had been determined using precision TIMS measurements and was reported as 210.0 Å^2^ [50]. Another aspect of trying C_60_ would be the fact that it was expected to form a molecular ion, thereby adding to the group of molecular ion species generated by APFD so far. In fact, the positive-ion APFD-TIMS analysis of [60]fullerene required an EHC of 0.16–0.17 A, which was the upper limit that could be set without destroying the emitter [31]. Under these conditions, the APFD spectrum showed a molecular ion peak at *m*/*z* 719.9988 that matched the [C_60_]^+•^ ion (calc. *m*/*z* 719.9995). The isotopic pattern of for [C_60_]^+•^ was in good agreement with the calculated values indicating that field ionization was the only ionization pathway in this case (Fig. 3). The peaks due to [C_60_]^+•^ ions were accompanied by minor signals at *m*/*z* 735.9941 that could be attributed to [C_60_O]^+•^ (calc. *m*/*z* 735.9944) and *m*/*z* 751.9888 due to [C_60_O_2_]^+•^ (calc. *m*/*z* 751.9893). The very low intensity of the peaks related to oxidized fullerene also demonstrated that oxidation did not play a relevant role here, even though C_60_ is prone to oxidation, and actually, was desorbing from a red hot emitter at the open atmosphere. In contrast to C_60_, the oxides also tended to form ions via protonation superimposing the isotopic patterns of their molecular ions.

In case of [60]fullerene, the TIMS analyzer was operated across a 1/*K*_0_ range of 0.60–1.40 at an inlet pressure of 2.7 mbar, but in (A) with an ion accumulation time of 100 ms and a ramp time of 400 ms and while in (B) with an ion accumulation time of 150 ms and a ramp time of 300 ms. The second settings were chosen to somewhat increase the molecular ion intensity at reduced TIMS resolution. After changing the TIMS parameters from (A) to (B), the TIMS analyzer was recalibrated using the ESI source before continuing APFD work. In either case, the CCS values of two sets of three measurements each were within 0.5% of the published CCS value for [C_60_]^+•^ of 210.0 Å^2^ [50]. Two additional runs taken from each setting are provided as Figs. S4 and S5 along with the tabulated data of all six runs.

**Fig. 4:**
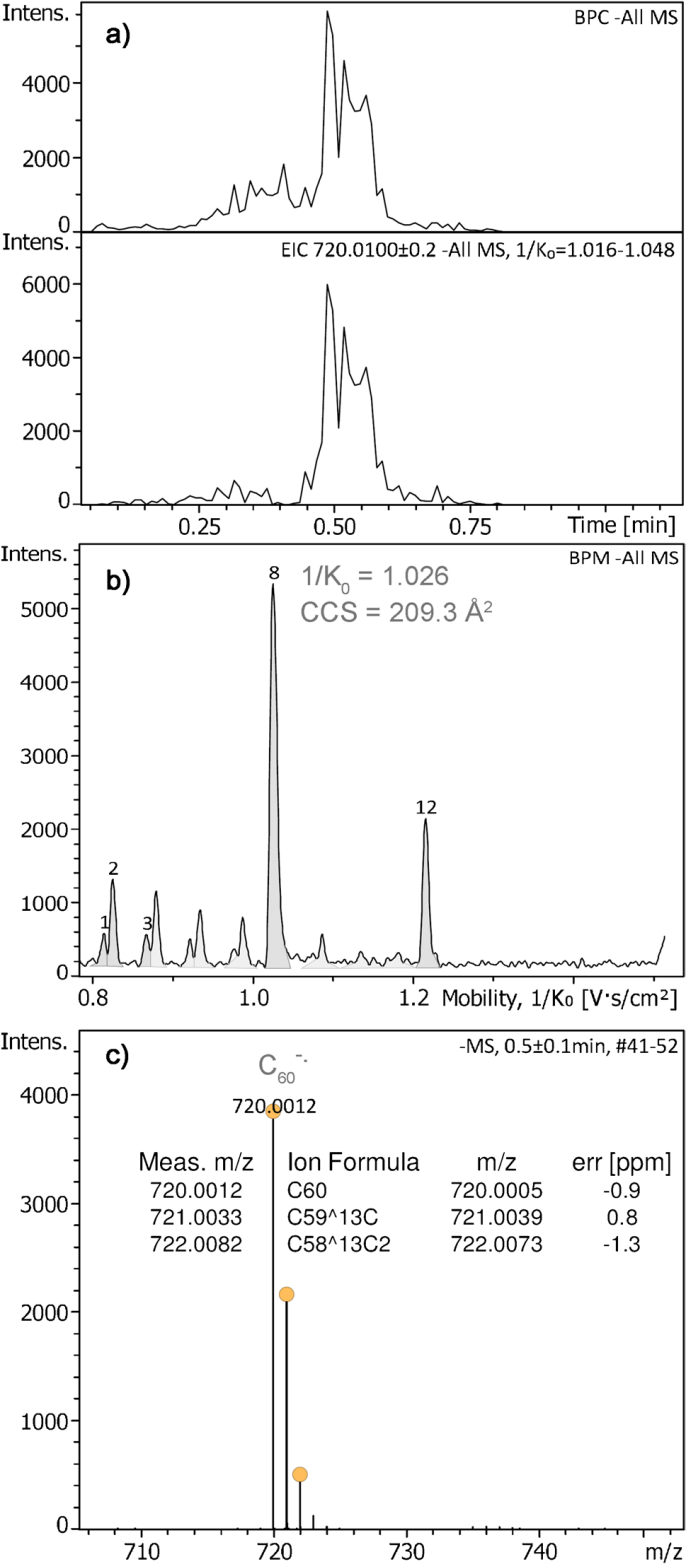
Negative-ion APFD-TIMS analysis of [60]fullerene. **a** Base peak chromatogram and extracted ion chromatogram showing the formation of [C_60_]^−•^ ions to occur at about 0.5 min when the EHC of 0.17 A was reached. **b** Base peak mobilogram with component 8 being the fullerene. **c** Mass spectrum showing the negative molecular ion accompanied by the carbon isotope ions. Other components were traces of residual anionic surfactant samples being ionized by far effectively than the fullerene

Negative-ion APFD-TIMS of [60]fullerene was also explored using 4.4 kV at the spray shield, a 1/K_0_ range of 0.75–1.45, an ion accumulation time of 50 ms, and a ramp time of 600 ms at an inlet pressure of 2.7 mbar. When an EHC of 0.17 A was reached, the sample delivered [C_60_]^−•^ ions, *m/z* 720.0012 (calc. *m/z* 720.0005), accompanied by the carbon isotope ions, the relative intensity of which indicated pure molecular anion formation (Fig. 4). The formation of [M]^−•^ ions is noteworthy as electron attachment generally unknown in negative-ion vacuum FD [46]. While in this experiment the focus was rather on feasibility than on CCS accuracy, the CCS value of 209.3 Å^2^ derived for [C_60_]^−•^ ions was still in reasonable agreement with the published value of 211.8 Å^2^ [50].

### Jeffamine M-2005

The positive-ion spectra of two different types of basic poly(propylene glycols) that are commercially available under the trade name Jeffamine were already known from previous work using either matrix-assisted laser desorption/ionization (MALDI) [51] or APFD [25]. Due to its basic amino end group, Jeffamine M-2005 delivered spectra exhibiting intensive signals corresponding to a series of protonated molecules that was spaced at Δ(*m*/*z*) = 58.0419 as expected for the C_3_H_6_O monomer unit. Here, Jeffamine M-2005 was reinvestigated with the TIMS analyzer in operation to explore the performance of the APFD-TIMS coupling with another type of analytes, and in particular with one of much wider *m*/*z* range and accordingly with components of lower mobility than PEG 300 or [60]fullerene. As with PEG 300 before, two sets of acquisitions were run with somewhat different settings. The first set (A) was acquired with a 1/*K*_0_ range of 0.70–2.00 at an inlet pressure of 2.5 mbar, an ion accumulation time of 50 ms, and a ramp time of 400 ms, while the second (B) was obtained with a 1/*K*_0_ range of 0.80–2.10 at an inlet pressure of 2.5 mbar, an ion accumulation time of 100 ms, and a ramp time of 500 ms.

The entire series comprising (i) *m*/*z* and TIMS calibration using Agilent TuneMix in ESI mode, (ii) acquisition of a sequence of three APFD-TIMS runs with the sample, (iii) a second *m*/*z* and TIMS calibration after a TIMS range adjustment, (iv) a sequence of three further APFD-TIMS runs, and (v) a final calibration in ESI mode to prepare for the next group of samples took less than 40 min. This also demonstrated the speed and ease of source switching from ESI to APFD and back. The full data set is provided in Figs. S6–S14.

**Fig. 5:**
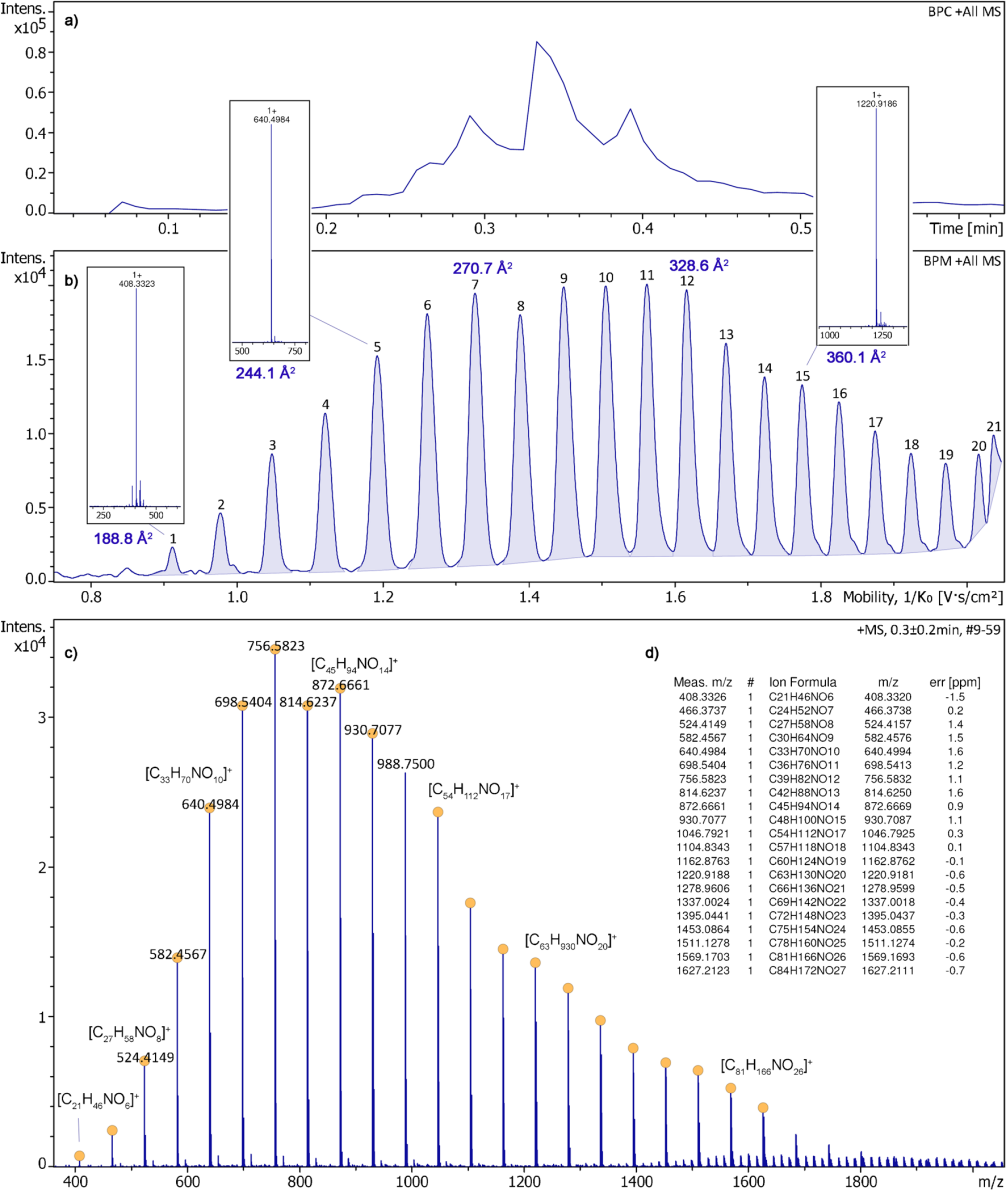
Positive-ion APFD-TIMS analysis of Jeffamine M-2005. **a** Base peak chromatogram (BPC) showing the ion current as affected by ramping the EHC up to 0.14 A. **b** Base peak mobilogram (BPM, setting B) revealing 21 components, some with CCS values annotated for orientation. The quality of the TIMS separation is exemplified by three inserts of compound spectra related to compounds #1, #5, and #15. **c** Mass spectrum as obtained by summing the desorption across the acquisition time with desorption (spectra #9–59) and **d** formula assignments to [M + H]^+^ ions based on accurate mass data. For this APFD analysis, the spray shield was set to –4500 V and the desolvation gas to 150 °C

While the EHC was manually increased up to 0.14 A, the [M + H]^+^ ions of Jeffamine M-2005 were created to deliver intensive spectra until most of the sample had been consumed (Fig. 5). The base peak mobilogram as obtained with setting B revealed 21 compounds that were well separated with the exception of the last pair at the upper limit of the mobility range. The ion series started with [C_21_H_46_NO_6_]^+^ at *m*/*z* 408.3326 (component #1) having a CCS of 188.8 Å^2^ and reached beyond component #19 being the last fully separated species, [C_75_H_154_NO_24_]^+^ at *m*/*z* 1453.0864, having a CCS of 399.0 Å^2^ (Fig. S13). The CCS values of all Jeffamine ions based on sets A and B were determined and compiled in Table S2 as these have not yet been published.

The APFD-TIMS mass spectrum obtained as the sum of spectra #9–59 very closely resembled the one published earlier without TIMS in terms of ions observed, *m*/*z* range covered, and intensity distribution [25, 31]. This work already indicated that with Jeffamine M-2005 and polystyrene APFD may cover ions at least up to *m*/*z* 1800. Here, in combination with TIMS, the 1/K_0_ range or upper 1/K_0_ limit strongly influences the upper *m*/*z* limit of the respective spectrum. Experience with FD tells us that, like with any other method, limits are strongly compound class-dependent, especially as high EHCs may induce thermal decomposition. In case of APFD, the upper *m/z* range admittedly may also be affected by oxidation or hydrolysis of the analyte particularly at high EHCs (> 0.15 A). However, as just proven by the results with [60]fullerene, the level of oxidation appears to be quite low, most probably due to the nitrogen desolvation gas flowing around the emitter.

### Polycyclic aromatic hydrocarbons

**Fig. 6:**
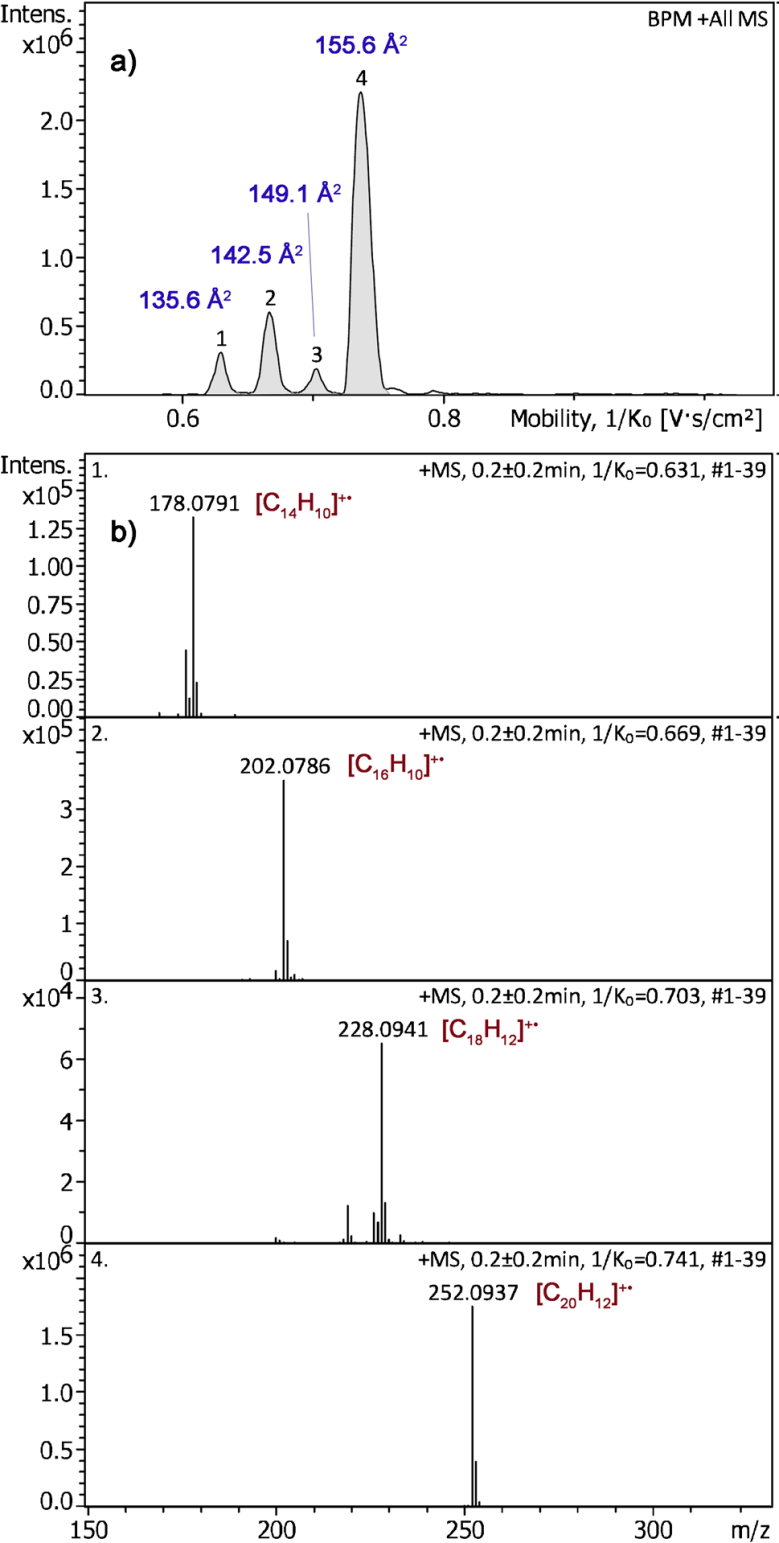
Positive-ion APFD-TIMS analysis of a mixture of four PAHs. **a** Base peak mobilogram (BPM, 1/*K*_0_ range of 0.50–1.00, inlet pressure 2.8 mbar, ion accum. 10 ms, ramp time 500 ms) showing the separation of anthracene (#1), fluoranthene (#2), triphenylene (#3), and benzo[a]pyrene (#4) with CCS values annotated to the peaks. **b** Partial component mass spectra showing the molecular ion regions and the formula assignments based on accurate mass data. For this APFD analysis, the spray shield was set to –4800 V and the desolvation gas to 150 °C

FI and FD are known to deliver molecular ions of polycyclic aromatic hydrocarbons (PAHs) [52], a compound class that is also of environmental concern [53]. Thus, this group of analytes was used to probe the capability of APFD-TIMS coupling in a potential field of application. Anthracene, fluoranthene, and benzo[a]pyrene had already been shown to form molecular ions in APFD via the field ionization process [26, 31]. A mixture of these plus triphenylene was transferred onto the emitter and analyzed by APFD-TIMS. Following advice on how to achieve good TIMS resolving power for low-mass, and thus, high-mobility ions [54, 55], the TIMS settings to acquire a 1/*K*_0_ range of 0.50–1.00 were adjusted to an inlet pressure of 2.8 mbar, an ion accumulation time of 10 ms, and a ramp time of 500 ms. The TIMS resolution achieved was sufficient to resolve all four components (Fig. 6). With the explorative nature of these experiments in mind, achieving the best accuracy of mass and CCS values was not a priority here. Still the CCS values determined for these PAH molecular ions were in satisfactory agreement with published data: anthracene 135.6 Å^2^ (Lit. [56] 133.1 Å^2^), fluoranthene 142.5 Å^2^ (Lit. [56] 138.4 Å^2^), triphenylene 149.1 Å^2^ (Lit. [56] 144.8 Å^2^), and benzo[a]pyrene 155.6 Å^2^ (Lit. [56] 151.2 Å^2^). A closer look at the molecular ion regions of the respective compound mass spectra revealed some loss of H_2_. These hydrogen losses yielded fragment ions having CCS values very close to those of the intact molecular ions, thereby causing some distortion of the TIMS signals. A comparison of the APFD spectra obtained with no TIMS in operation showed an increase of hydrogen losses, most probably due to some collision-induced dissociation of the M^+•^ ions during the elongated period of trapping [57]. With TIMS in operation and using the above settings, the relative intensity of the [M–H_2_]^+•^ ion of anthracene rose from about 7 to 19%, for example. The formation of these fragment ions certainly had a negative effect on the accuracy of the CCS values in this particular case. Nonetheless, this set of experiments indicated the potential of APFD-TIMS for PAH analysis, and therefore, the limits of detection (LODs) were determined for benzo[a]pyrene and fluoranthene using the above TIMS range settings. The intensities of the molecular ion peaks of each compound were determined three times per sample load and averages of the three runs were taken as a measure of intensity. An intensity of a few hundred counts was assumed to represent a good measure for a still useable spectrum. Thus, the LODs were determined as ≈ 100 pg for fluoranthene and as < 1 pg for benzo[a]pyrene (Table S3 and Fig. S15). The by two orders of magnitude lower LOD of benzo[a]pyrene is in line with its by 0.8 eV lower ionization energy (7.12 eV vs. 7.9 eV [26]).

## Conclusions

A recently developed APFD ion source was used on a trapped ion mobility-quadrupole-time-of-flight (TIMS-Q-TOF) instrument (Bruker timsTOFflex) and trapped ion mobility separation of the ions delivered by APFD was examined. The operation and performance of this new APFD-TIMS-MS coupling were explored. APFD-TIMS-MS not only allowed for the separation of individual components of oligomers forming either [M + H]^+^ or [M + Na]^+^ ions but also of compounds forming molecular ions, M^+•^, that are more prone to fragmentation. Even-electron ion species were obtained from poly(ethylene glycol) and an amine-terminated poly(propylene glycol) while [60]fullerene and a mixture of four polycyclic aromatic hydrocarbons yielded molecular ions via field ionization. Even though desorbed from a red hot emitter and having a tendency to undergo oxidation, in the APFD spectrum of C_60_, there was only a minor amount of fullerene oxides to be observed. By comparison to published CCS data in case of PEG and [60]fullerene, APFD-TIMS-MS was proven to deliver accurate CCS values. APFD-TIMS-MS was then applied for the determination of CCS values of the amine-terminated poly(propylene glycol). Finally, a potential application in PAH mixture analysis was investigated and LODs of two representative compounds were determined. Overall, APFD-TIMS-MS turned out to offer robust operation and new analytical possibilities.

### Supplementary Information

Below is the link to the electronic supplementary material.

Supplementary file1 (PDF 7722 KB)
