# Supplementary material for: Atmospheric pressure field desorption-trapped ion mobility-mass spectrometry coupling
Source: Anal Bioanal Chem. 2024 Apr 8;416(14):3313–23. doi: 10.1007/s00216-024-05282-0 (PMC11106181; doi:10.1007/s00216-024-05282-0)

Atmospheric pressure field desorption-trapped ion mobility-mass spectrometry coupling

Supplementary Data

Jürgen H. Gross

Institute of Organic Chemistry

Heidelberg University

Im Neuenheimer Feld 270

69120 Heidelberg

Germany

* Send correspondence to Jürgen H. Gross

ORCID 0000-0003-0748-2535

email juergen.gross@oci.uni-heidelberg.de

phone +49/6221/54-8409

Figs. S1 and S2. These photographs show the probe mounting stage while the frame is mounted to the AP interface of the Bruker timsTOFflex instrument. Fig. S1 (*top*) shows the emitter in retracted position as to allow for emitter loading or emitter replacement and Fig. S2 (*bottom*) displays it in operational position with the emitter at 2.0 mm distance to the spray shield. The knurled brass screws at the right and top of the emitter mounting stage allow for a fine *x*,*y*-adjustment of the emitter position, which may only be required when the entire unit has just been mounted. The USB microscope always moves with the emitter mounting stage, and thus, provides an image of the emitter at any time during operation.

S1

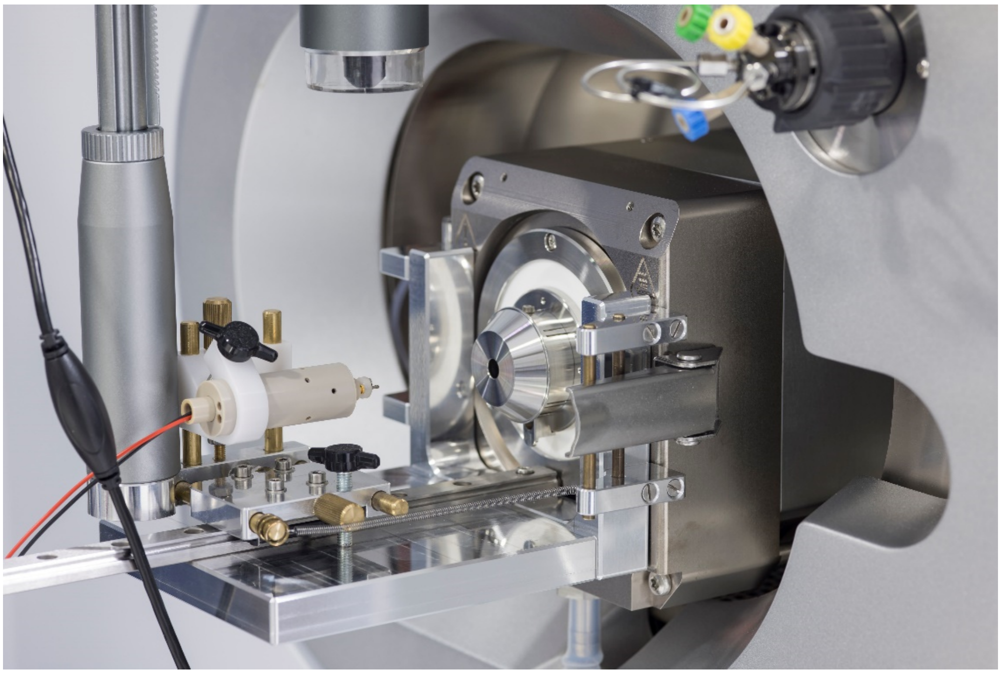

S2

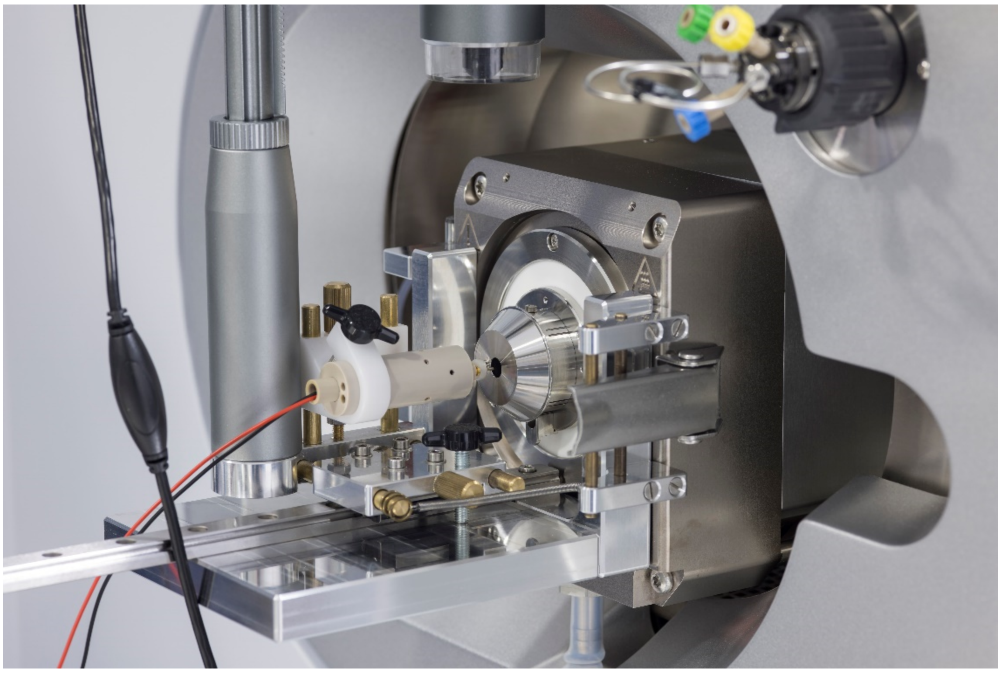

Fig. S3. Evaluation of CCS values obtained from a sequence of three APFD-TIMS analyses of PEG 300. This figure shows the data of the third run, while that of the first is provided as Fig. 2 in the article.

Sample PEG300 Instrument timsTOF fleX 1859745.20462

Comment PEG 300, 1ug, APFD 4300V 4l 150C EHC<120mA, TIMS 2.7 mbar, custom 0.60-1.40, ramp 400, accum 100 ms, dutycycle 25%, kalib TIMS

Acquisition Parameter

Source Type	ESI	Ion Polarity	Positive	Set Nebulizer	0.0 Bar
Scan Begin	100 m/z	Set Capillary	4300 V	Set Dry Heater	150 °C
Scan End	2000 m/z	Set Multipole RF	300.0 Vpp	Set Dry Gas	4.0 l/min
		Set Collision Cell RF	650.0 Vpp	ICC active	Off
		IMS Active	On	ICC Target	2000000 cts.
		IMS Collision Cell In	300.0 V		

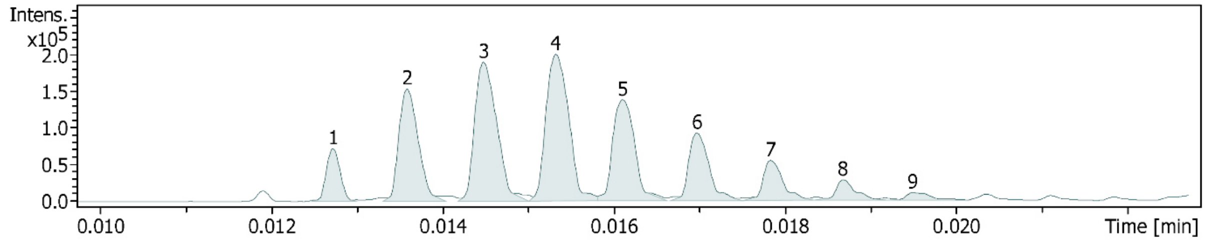

#	RT [min]	Area	Int. Type	I	Trace	Max. m/z	FWHM [min]	CCS [Å²]	Mobility 1/K0	Resolution 1/K0
1	-0.0		Mobilogram		BPM +All MS	305.1564		159.7	0.763	70.300
2	-0.0		Mobilogram		BPM +All MS	349.1828		169.7	0.815	54.992
3	-0.0		Mobilogram		BPM +All MS	393.2095		180.0	0.869	50.243
4	-0.0		Mobilogram		BPM +All MS	437.2353		189.9	0.919	51.025
5	-0.0		Mobilogram		BPM +All MS	481.2613		199.0	0.966	56.762
6	-0.0		Mobilogram		BPM +All MS	525.2874		209.3	1.018	67.154
7	-0.0		Mobilogram		BPM +All MS	569.3134		219.4	1.070	75.971
8	-0.0		Mobilogram		BPM +All MS	613.3397		229.4	1.121	90.502
9	-0.0		Mobilogram		BPM +All MS	657.3662		239.1	1.169	66.894

Tab. S1. TIMS data of compounds #1 to #9 taken from runs #1 to #3 and comparison of the average CCS values to the reference CCS values from the literature*.

			CCS [Å2]					
Com-pound No.	Molecule	Nominal m/z	Run #1	Run #2	Run #3	Average CCS	CCS from Lit.*	Delta CCS [%]
1	6mer	305	159.5	159.9	159.7	159.7	161	0.8
2	7mer	349	169.5	169.8	169.7	169.7	168	-1.0
3	8mer	393	180.1	180.2	180.0	180.1	183	1.6
4	9mer	437	189.8	189.9	189.9	189.9	187	-1.6
5	10mer	481	198.9	199.1	199.0	199.0	197	-1.0
6	11mer	525	209.4	209.7	209.0	209.4	208	-0.5
7	12mer	569	219.7	220.0	219.4	219.7	219	-0.2
8	13mer	613	229.8	230.0	229.4	229.7	233	1.5
9	14mer	657	239.4	239.6	239.1	239.4	239	0.0

* Fiebig L and Laux R. A collision cross section and exact ion mass database of the formulation constituents polyethylene glycol 400 and polysorbate 80. *International Journal for Ion Mobility Spectrometry* 2016; 19: 131-137; DOI: 10.1007/s12127-016-0195-2.

Figs. S4 and S5. CCS determination of C_{60} by APFD-TIMS analysis. The TIMS analyzer was operated across a $1/K_0$ range of 0.60–1.40 at 2.7 mbar, but in A) (*top*) with ion accumulation at 100 ms and ramp time 400 ms while in B) (*bottom*) with ion accumulation of 150 ms and ramp time 300 ms. The second settings were chosen to increase the molecular ion intensity at a reduced TIMS resolution (from 104 to 61). The table below compiles all six CCS runs.

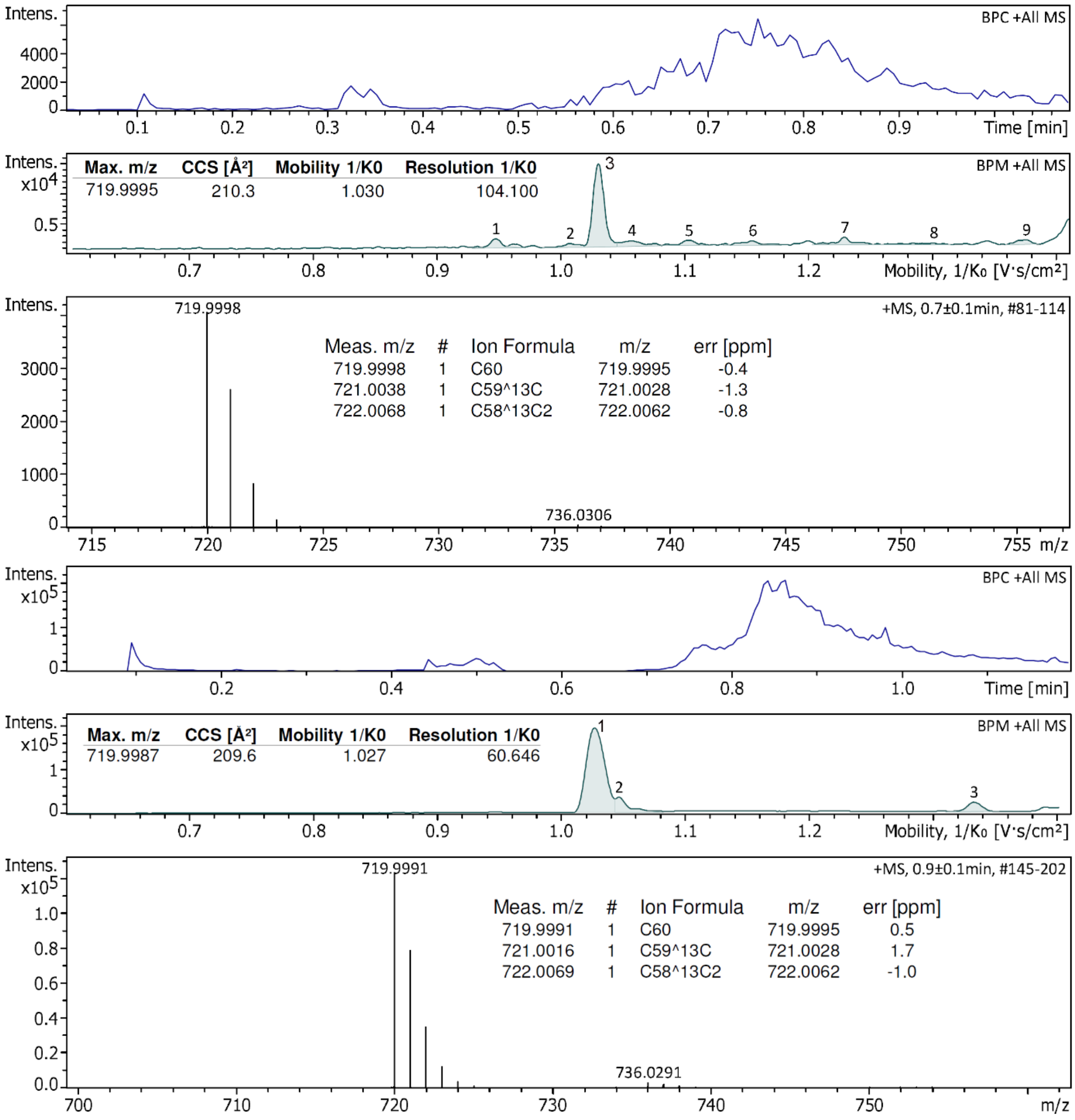

Run No.	CCS [Å2]	CCS Ref.*	Delta CCS [%]	
A1	209.6	210	0.19	100 ms accum., 400 ms ramp, 1/Ko range 0.60 to 1.40, 2.7 mbar
A2	210.3	210	-0.14	
A3	211.1	210	-0.52	
B1	209.8	210	0.10	150 ms accum., 300 ms ramp, 1/Ko range 0.60 to 1.40, 2.7 mbar
B2	209.6	210	0.19	
B3	209.9	210	0.05	

*Weis P, Hennrich F, Fischer R, et al. *Phys. Chem. Chem. Phys.* 2019; 21: 18877-18892; DOI: 10.1039/C9CP03326B.

Fig. S6–S14. Positive-ion APFD-TIMS analysis of Jeffamine M-2005. On the following pages, a complete series comprising i) *m/z* and TIMS calibration using Agilent TuneMix in ESI mode, ii) sequence of three APFD-TIMS runs with the sample, iii) a second *m/z* and TIMS calibration after a range adjustment, iv) sequence of three further APFD-TIMS runs, and v) a final calibration to prepare for the next group of samples are shown, in order to demonstrate the speed and ease of source switching from ESI to APFD and also to deliver the full data set. Actual acquisition times are annotated in the upper right corner of each figure. Starting time of first run at 2.49 pm and start of calibration afterwards at 3.26 pm.

S6. ESI mode for *m/z* and TIMS calibration.

Analysis Info

Analysis	D:\Projekte\APFD_TIMS_2024\DataTimsTOF\tims8915_2.d	Acquisition Date	22.01.2024 14:29:10
Method	ESIpos_100-2500_detect.m	Operator	TOF-User
Sample	TuneMix	Instrument	timsTOF fleX 1859745.20462
Comment	TuneMix TIMS 2.7 mbar, custom 0.70-2.00, ramp 400, accum 50 ms, dutycycle 12.5%, kalib TIMS		

Acquisition Parameter

Source Type	ESI	Ion Polarity	Positive	Set Nebulizer	0.5 Bar
Scan Begin	100 m/z	Set Capillary	3800 V	Set Dry Heater	220 °C
Scan End	2500 m/z	Set Multipole RF	400.0 Vpp	Set Dry Gas	4.0 l/min
		Set Collision Cell RF	1200.0 Vpp	ICC active	Off
		IMS Active	On	ICC Target	2000000 cts.
		IMS Collision Cell In	300.0 V		

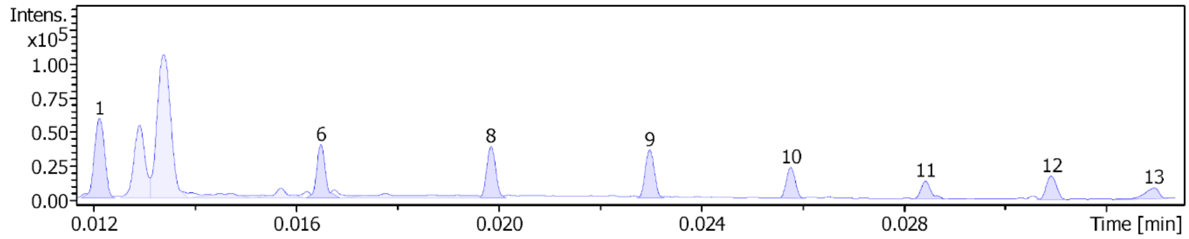

#	RT [min]	Area	Int. Type	I	Trace	Max. m/z	FWHM [min]	CCS [Å²]	Mobility 1/K0	Resolution 1/K0
1	-0.0		Mobilogram		BPM +All MS	322.0475		151.9	0.728	53.997
6	-0.0		Mobilogram		BPM +All MS	622.0279		202.6	0.990	90.600
8	-0.0		Mobilogram		BPM +All MS	922.0102		242.1	1.191	101.689
9	-0.0		Mobilogram		BPM +All MS	1221.9918		279.3	1.379	114.088
10	-0.0		Mobilogram		BPM +All MS	1521.9743		312.4	1.546	131.031
11	-0.0		Mobilogram		BPM +All MS	1821.9544		344.1	1.705	142.668
12	-0.0		Mobilogram		BPM +All MS	2121.9316		373.8	1.854	135.808
13	-0.0		Mobilogram		BPM +All MS	2421.9095		398.0	1.976	117.176

Instrument calibration

Date:	22.01.2024 14:26:25
Polarity:	Positive
Calibration spectrum:	<unknown>
Reference mass list:	Tuning Mix ES-TOF (ESI)
Calibration mode:	Enhanced Quadratic
Standard deviation:	0.442 ppm

Reference m/z	Resulting m/z	Intensity	Error [ppm]
322.0481	322.0481	80345	-0.143
622.0290	622.0293	22040	0.538
922.0098	922.0095	19130	-0.360
1221.9906	1221.9901	15956	-0.406
1521.9715	1521.9716	10435	0.086
1821.9523	1821.9535	6174	0.653
2121.9331	2121.9324	7199	-0.368

Instrument mobility calibration

Date:	22.01.2024 14:28:49
Polarity:	Positive
Calibration mobilogram:	<unknown>
Reference mass list:	Tuning Mix ES-TOF
Standard deviation:	4613.764%

Reference mobility	Resulting mobility	Intensity	Error [%]
0.732	0.730	4131824	-0.206
0.985	0.989	3401675	0.466
1.190	1.192	3165304	0.231
1.382	1.381	2898980	-0.087
1.556	1.548	2197769	-0.494

S7. APFD-TIMS analysis of Jeffamine M-2005, #1.

Analysis Info

Analysis	D:\Projekte\APFD_TIMS_2024\DataTimsTOF\tims8916_3.d
Method	APFD_pos_100-2500_custom.m
Sample	TuneMix
Comment	Jeffamin M2005, 1uL, APFD 4500V 4l 150C EHC<100mA,TuneMix TIMS 2.5 mbar, custom 0.70-2.00, ramp 400, accum 50 ms, dutycycle 12.5%, kalib TIMS

Acquisition Date	22.01.2024 14:42:33
Operator	TOF-User
Instrument	timsTOF fleX 1859745.20462

Acquisition Parameter

Source Type	ESI	Ion Polarity	Positive	Set Nebulizer	0.0 Bar
Scan Begin	100 m/z	Set Capillary	4500 V	Set Dry Heater	150 °C
Scan End	2500 m/z	Set Multipole RF	400.0 Vpp	Set Dry Gas	4.0 l/min
		Set Collision Cell RF	1200.0 Vpp	ICC active	Off
		IMS Active	On	ICC Target	2000000 cts.
		IMS Collision Cell In	300.0 V		

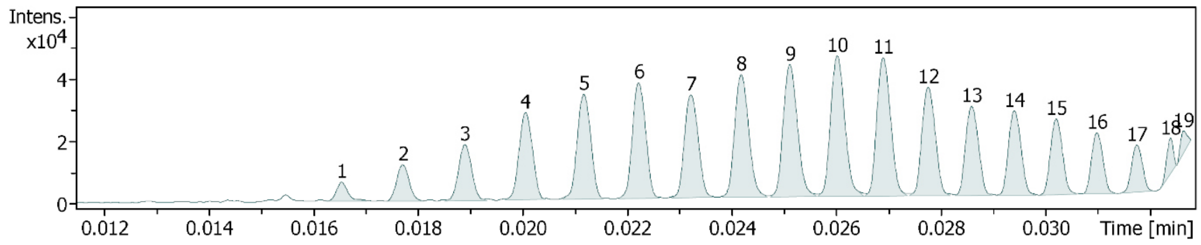

#	RT [min]	Area	Int. Type	I	Trace	Max. m/z	FWHM [min]	CCS [Å²]	Mobility 1/K0	Resolution 1/K0
1	-0.0		Mobilogram		BPM +All MS	466.3724		204.7	0.993	74.378
2	-0.0		Mobilogram		BPM +All MS	524.4141		218.4	1.063	67.316
3	-0.0		Mobilogram		BPM +All MS	582.4559		232.5	1.134	68.076
4	-0.0		Mobilogram		BPM +All MS	640.4976		246.2	1.204	69.614
5	-0.0		Mobilogram		BPM +All MS	698.5399		259.4	1.270	72.947
6	-0.0		Mobilogram		BPM +All MS	756.5820		271.9	1.333	76.768
7	-0.0		Mobilogram		BPM +All MS	814.6239		283.8	1.393	78.991
8	-0.0		Mobilogram		BPM +All MS	872.6656		295.2	1.451	82.593
9	-0.0		Mobilogram		BPM +All MS	930.7071		306.2	1.507	84.238
10	-0.0		Mobilogram		BPM +All MS	988.7486		317.0	1.561	88.175
11	-0.0		Mobilogram		BPM +All MS	1046.7903		327.4	1.613	91.693
12	-0.0		Mobilogram		BPM +All MS	1104.8322		337.6	1.665	96.948
13	-0.0		Mobilogram		BPM +All MS	1162.8738		347.6	1.715	101.865
14	-0.0		Mobilogram		BPM +All MS	1220.9154		357.3	1.764	108.625
15	-0.0		Mobilogram		BPM +All MS	1278.9566		366.8	1.812	117.757
16	-0.0		Mobilogram		BPM +All MS	1336.9984		376.1	1.859	125.950
17	-0.0		Mobilogram		BPM +All MS	1395.0402		385.2	1.904	134.359
18	-0.0		Mobilogram		BPM +All MS	1453.0818		392.8	1.943	229.640
19	-0.0		Mobilogram		BPM +All MS	1511.1234		395.7	1.958	164.730

S8. APFD-TIMS analysis of Jeffamine M-2005, #2.

Analysis Info

Analysis	D:\Projekte\APFD_TIMS_2024\DataTimsTOF\tims8917_1.d
Method	APFD_pos_100-2500_custom.m
Sample	TuneMix
Comment	Jeffamin M2005, 1uL, APFD 4500V+500V 4l 150C EHC<140mA,TuneMix TIMS 2.5 mbar, custom 0.70-2.00, ramp 400, accum 50 ms, dutycycle 12.5%, kalib TIMS

Acquisition Date 22.01.2024 14:50:46

Operator TOF-User

Instrument timsTOF fleX 1859745.20462

Acquisition Parameter

Source Type	ESI	Ion Polarity	Positive	Set Nebulizer	0.0 Bar
Scan Begin	100 m/z	Set Capillary	4500 V	Set Dry Heater	150 °C
Scan End	2500 m/z	Set Multipole RF	400.0 Vpp	Set Dry Gas	4.0 l/min
		Set Collision Cell RF	1200.0 Vpp	ICC active	Off
		IMS Active	On	ICC Target	2000000 cts.
		IMS Collision Cell In	300.0 V		

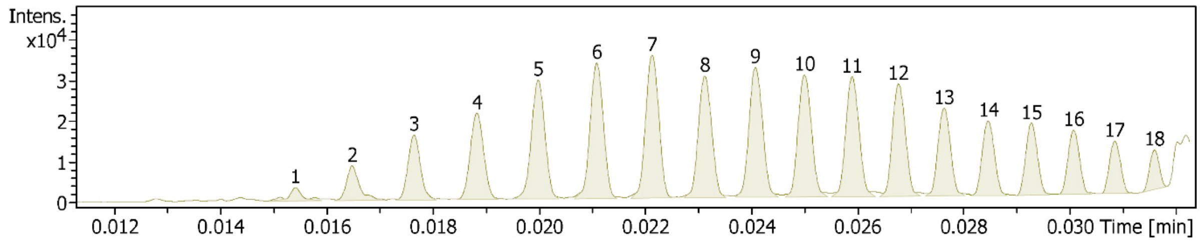

#	RT [min]	Area	Int. Type	I	Trace	Max. m/z	FWHM [min]	CCS [Å²]	Mobility 1/K0	Resolution 1/K0
1	-0.0		Mobilogram		BPM +All MS	408.3313		191.5	0.925	76.764
2	-0.0		Mobilogram		BPM +All MS	466.3728		204.0	0.989	69.802
3	-0.0		Mobilogram		BPM +All MS	524.4145		217.7	1.059	69.843
4	-0.0		Mobilogram		BPM +All MS	582.4562		231.7	1.130	67.608
5	-0.0		Mobilogram		BPM +All MS	640.4979		245.3	1.199	72.360
6	-0.0		Mobilogram		BPM +All MS	698.5399		258.4	1.265	76.964
7	-0.0		Mobilogram		BPM +All MS	756.5819		270.8	1.328	80.561
8	-0.0		Mobilogram		BPM +All MS	814.6238		282.6	1.388	82.472
9	-0.0		Mobilogram		BPM +All MS	872.6654		293.9	1.445	85.444
10	-0.0		Mobilogram		BPM +All MS	930.7072		304.8	1.500	89.394
11	-0.0		Mobilogram		BPM +All MS	988.7490		315.5	1.554	93.883
12	-0.0		Mobilogram		BPM +All MS	1046.7909		325.9	1.606	98.261
13	-0.0		Mobilogram		BPM +All MS	1104.8326		336.1	1.658	103.175
14	-0.0		Mobilogram		BPM +All MS	1162.8742		346.0	1.708	110.060
15	-0.0		Mobilogram		BPM +All MS	1220.9161		355.7	1.756	119.747
16	-0.0		Mobilogram		BPM +All MS	1278.9579		365.2	1.804	123.364
17	-0.0		Mobilogram		BPM +All MS	1336.9995		374.4	1.850	129.289
18	-0.0		Mobilogram		BPM +All MS	1395.0414		383.3	1.895	152.490

S9. APFD-TIMS analysis of Jeffamine M-2005, #3.

Analysis Info

Acquisition Date 22.01.2024 14:55:57

Analysis D:\Projekte\APFD_TIMS_2024\DataTimsTOF\tims8917_2.d

Method APFD_pos_100-2500_custom.m

Sample TuneMix

Operator TOF-User

Instrument timsTOF fleX 1859745.20462

Comment Jeffamin M2005, 1uL, APFD 4500V+500V 4l 150C EHC<140mA,TuneMix TIMS 2.5 mbar, custom 0.70-2.00, ramp 400, accum 50 ms, dutycycle 12.5%, kalib TIMS

Acquisition Parameter

Source Type	ESI	Ion Polarity	Positive	Set Nebulizer	0.0 Bar
Scan Begin	100 m/z	Set Capillary	4500 V	Set Dry Heater	150 °C
Scan End	2500 m/z	Set Multipole RF	400.0 Vpp	Set Dry Gas	4.0 l/min
		Set Collision Cell RF	1200.0 Vpp	ICC active	Off
		IMS Active	On	ICC Target	2000000 cts.
		IMS Collision Cell In	300.0 V		

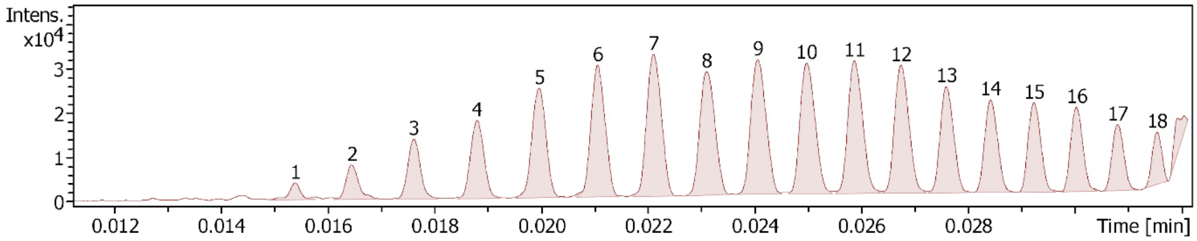

#	RT [min]	Area	Int. Type	I	Trace	Max. m/z	FWHM [min]	CCS [Å²]	Mobility 1/K0	Resolution 1/K0
1	-0.0		Mobilogram		BPM +All MS	408.3309		191.2	0.924	76.168
2	-0.0		Mobilogram		BPM +All MS	466.3725		203.5	0.987	67.188
3	-0.0		Mobilogram		BPM +All MS	524.4142		217.3	1.057	69.567
4	-0.0		Mobilogram		BPM +All MS	582.4559		231.3	1.128	67.058
5	-0.0		Mobilogram		BPM +All MS	640.4976		245.0	1.198	68.261
6	-0.0		Mobilogram		BPM +All MS	698.5395		258.0	1.263	71.138
7	-0.0		Mobilogram		BPM +All MS	756.5811		270.5	1.326	71.468
8	-0.0		Mobilogram		BPM +All MS	814.6226		282.3	1.386	73.016
9	-0.0		Mobilogram		BPM +All MS	872.6647		293.7	1.444	75.182
10	-0.0		Mobilogram		BPM +All MS	930.7063		304.5	1.498	79.053
11	-0.0		Mobilogram		BPM +All MS	988.7482		315.1	1.552	84.775
12	-0.0		Mobilogram		BPM +All MS	1046.7902		325.5	1.604	89.633
13	-0.0		Mobilogram		BPM +All MS	1104.8321		335.6	1.655	96.659
14	-0.0		Mobilogram		BPM +All MS	1162.8734		345.5	1.705	104.233
15	-0.0		Mobilogram		BPM +All MS	1220.9151		355.2	1.754	109.430
16	-0.0		Mobilogram		BPM +All MS	1278.9569		364.7	1.801	118.530
17	-0.0		Mobilogram		BPM +All MS	1336.9985		373.9	1.848	126.149
18	-0.0		Mobilogram		BPM +All MS	1395.0407		382.7	1.892	151.778
19	-0.0		Mobilogram		BPM +All MS	1453.0822		388.7	1.923	157.601

S10. ESI mode for second *m/z* and TIMS calibration after adjustment of TIMS settings.

Analysis Info

Analysis	D:\Projekte\APFD_TIMS_2024\DataTimsTOF\tims8918_1.d	Acquisition Date	22.01.2024 15:05:48
Method	APFD_pos_100-2500_custom.m	Operator	TOF-User
Sample	TuneMix	Instrument	timsTOF fleX 1859745.20462
Comment	TuneMix TIMS 2.5 mbar, custom 0.80-2.10, ramp 500, accum 100 ms, dutycycle 20%, kalib TIMS		

Acquisition Parameter

Source Type	ESI	Ion Polarity	Positive	Set Nebulizer	0.5 Bar
Scan Begin	100 m/z	Set Capillary	3800 V	Set Dry Heater	150 °C
Scan End	2500 m/z	Set Multipole RF	400.0 Vpp	Set Dry Gas	4.0 l/min
		Set Collision Cell RF	1200.0 Vpp	ICC active	Off
		IMS Active	On	ICC Target	2000000 cts.
		IMS Collision Cell In	300.0 V		

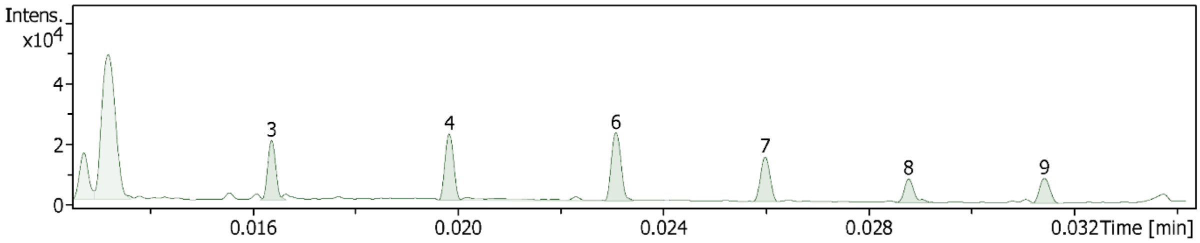

#	RT [min]	Area	Int. Type	I	Trace	Max. m/z	FWHM [min]	CCS [Å²]	Mobility 1/K0	Resolution 1/K0
3	-0.0		Mobilogram		BPM +All MS	622.0281		201.1	0.983	93.231
4	-0.0		Mobilogram		BPM +All MS	922.0095		241.9	1.190	104.936
6	-0.0		Mobilogram		BPM +All MS	1221.9913		280.4	1.385	111.015
7	-0.0		Mobilogram		BPM +All MS	1521.9726		315.0	1.559	124.857
8	-0.0		Mobilogram		BPM +All MS	1821.9522		348.4	1.726	145.873
9	-0.0		Mobilogram		BPM +All MS	2121.9300		380.0	1.885	139.961

Instrument calibration

Date:	22.01.2024 15:03:13
Polarity:	Positive
Calibration spectrum:	<unknown>
Reference mass list:	Tuning Mix ES-TOF (ESI)
Calibration mode:	Enhanced Quadratic
Standard deviation:	0.316 ppm

Reference m/z	Resulting m/z	Intensity	Error [ppm]
622.0290	622.0290	12160	0.033
922.0098	922.0097	11318	-0.083
1221.9906	1221.9905	9171	-0.100
1521.9715	1521.9722	5480	0.473
1821.9523	1821.9514	3247	-0.481
2121.9331	2121.9335	3944	0.157

Instrument mobility calibration

Date:	22.01.2024 15:04:00
Polarity:	Positive
Calibration mobilogram:	<unknown>
Reference mass list:	Tuning Mix ES-TOF (ESI)
Standard deviation:	3346.083%

Reference mobility	Resulting mobility	Intensity	Error [%]
0.985	0.983	1716468	-0.172
1.190	1.190	1881634	0.074
1.382	1.386	1840667	0.286
1.556	1.560	1311445	0.301
1.729	1.726	858440	-0.130
1.884	1.886	836120	0.099
2.030	2.019	427833	-0.530

S11. APFD-TIMS analysis of Jeffamine M-2005, #4

Analysis Info

Acquisition Date 22.01.2024 15:09:02

Analysis	D:\Projekte\APFD_TIMS_2024\DataTimsTOF\tims8919_1.d		
Method	APFD_pos_100-2500_custom.m	Operator	TOF-User
Sample	Jeffamine	Instrument	timsTOF fleX 1859745.20462
Comment	Jeffamin M-2005, 1uL, APFD 4500V+500V 4l 150C EHC<140mATuneMix TIMS 2.5 mbar, custom 0.80-2.10, ramp 500, accum 100 ms, dutycycle 20%, kalib TIMS		

Acquisition Parameter

Source Type	ESI	Ion Polarity	Positive	Set Nebulizer	0.0 Bar
Scan Begin	100 m/z	Set Capillary	4500 V	Set Dry Heater	150 °C
Scan End	2500 m/z	Set Multipole RF	400.0 Vpp	Set Dry Gas	4.0 l/min
		Set Collision Cell RF	1200.0 Vpp	ICC active	Off
		IMS Active	On	ICC Target	2000000 cts.
		IMS Collision Cell In	300.0 V		

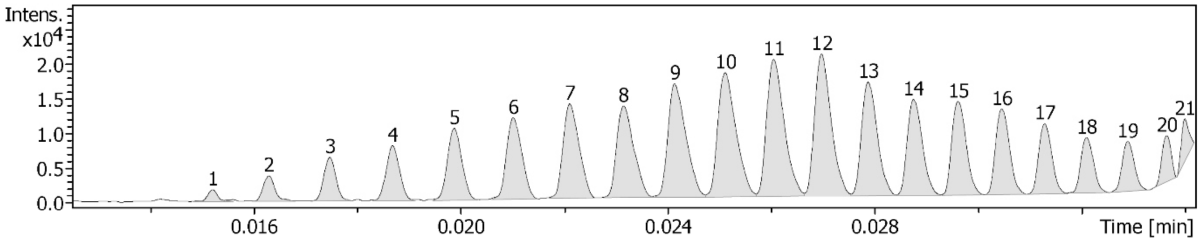

#	RT [min]	Area	Int. Type	I	Trace	Max. m/z	FWHM [min]	CCS [Å²]	Mobility 1/K0	Resolution 1/K0
1	-0.0		Mobilogram		BPM +All MS	408.3322		188.9	0.913	76.668
2	-0.0		Mobilogram		BPM +All MS	466.3739		201.6	0.978	67.720
3	-0.0		Mobilogram		BPM +All MS	524.4151		215.5	1.049	67.870
4	-0.0		Mobilogram		BPM +All MS	582.4566		229.9	1.121	66.016
5	-0.0		Mobilogram		BPM +All MS	640.4985		244.0	1.193	65.301
6	-0.0		Mobilogram		BPM +All MS	698.5406		257.6	1.261	62.891
7	-0.0		Mobilogram		BPM +All MS	756.5826		270.5	1.327	63.942
8	-0.0		Mobilogram		BPM +All MS	814.6245		282.8	1.389	62.848
9	-0.0		Mobilogram		BPM +All MS	872.6662		294.6	1.448	63.611
10	-0.0		Mobilogram		BPM +All MS	930.7077		306.2	1.507	65.222
11	-0.0		Mobilogram		BPM +All MS	988.7496		317.3	1.563	71.247
12	-0.0		Mobilogram		BPM +All MS	1046.7913		328.4	1.618	77.847
13	-0.0		Mobilogram		BPM +All MS	1104.8333		339.1	1.672	83.542
14	-0.0		Mobilogram		BPM +All MS	1162.8759		349.5	1.725	89.493
15	-0.0		Mobilogram		BPM +All MS	1220.9179		359.8	1.777	96.084
16	-0.0		Mobilogram		BPM +All MS	1278.9599		369.9	1.827	102.223
17	-0.0		Mobilogram		BPM +All MS	1337.0018		379.7	1.877	111.093
18	-0.0		Mobilogram		BPM +All MS	1395.0434		389.4	1.925	117.298
19	-0.0		Mobilogram		BPM +All MS	1453.0850		398.8	1.973	126.814
20	-0.0		Mobilogram		BPM +All MS	1511.1269		407.8	2.018	164.824
21	-0.0		Mobilogram		BPM +All MS	1569.1698		411.9	2.039	156.798

S12. APFD-TIMS analysis of Jeffamine M-2005, #5

Analysis Info

Acquisition Date 22.01.2024 15:14:32

Analysis D:\Projekte\APFD_TIMS_2024\DataTimsTOF\tims8919_2.d
Method APFD_pos_100-2500_custom.m
Sample Jeffamine
Comment Jeffamin M-2005, 1uL, APFD 4500V+500V 4l 150C EHC<140mATuneMix TIMS 2.5 mbar, custom 0.80-2.10, ramp 500, accum 100 ms, dutycycle 20%, kalib TIMS

Operator TOF-User
Instrument timsTOF fleX 1859745.20462

Acquisition Parameter

Source Type	ESI	Ion Polarity	Positive	Set Nebulizer	0.0 Bar
Scan Begin	100 m/z	Set Capillary	4500 V	Set Dry Heater	150 °C
Scan End	2500 m/z	Set Multipole RF	400.0 Vpp	Set Dry Gas	4.0 l/min
		Set Collision Cell RF	1200.0 Vpp	ICC active	Off
		IMS Active	On	ICC Target	2000000 cts.
		IMS Collision Cell In	300.0 V		

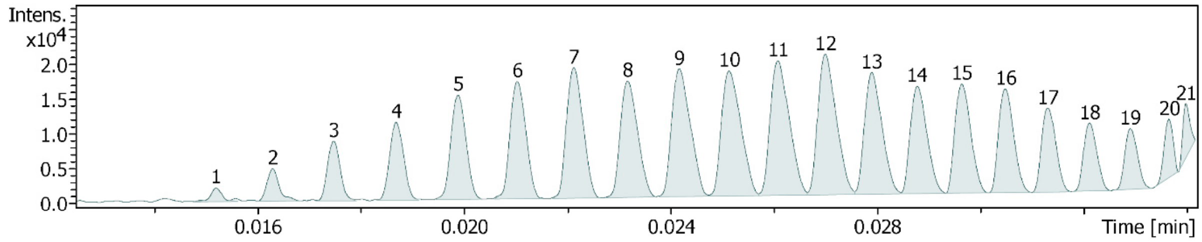

#	RT [min]	Area	Int. Type	I	Trace	Max. m/z	FWHM [min]	CCS [Å²]	Mobility 1/K0	Resolution 1/K0
1	-0.0		Mobilogram		BPM +All MS	408.3325		188.8	0.912	69.670
2	-0.0		Mobilogram		BPM +All MS	466.3736		201.6	0.978	66.769
3	-0.0		Mobilogram		BPM +All MS	524.4150		215.6	1.049	64.153
4	-0.0		Mobilogram		BPM +All MS	582.4566		229.9	1.121	61.610
5	-0.0		Mobilogram		BPM +All MS	640.4983		244.1	1.193	60.910
6	-0.0		Mobilogram		BPM +All MS	698.5399		257.7	1.262	60.756
7	-0.0		Mobilogram		BPM +All MS	756.5816		270.7	1.328	61.258
8	-0.0		Mobilogram		BPM +All MS	814.6231		283.1	1.390	60.273
9	-0.0		Mobilogram		BPM +All MS	872.6654		295.0	1.450	59.407
10	-0.0		Mobilogram		BPM +All MS	930.7076		306.4	1.508	59.933
11	-0.0		Mobilogram		BPM +All MS	988.7498		317.7	1.565	62.934
12	-0.0		Mobilogram		BPM +All MS	1046.7919		328.6	1.619	68.807
13	-0.0		Mobilogram		BPM +All MS	1104.8340		339.3	1.673	74.008
14	-0.0		Mobilogram		BPM +All MS	1162.8761		349.8	1.726	80.321
15	-0.0		Mobilogram		BPM +All MS	1220.9182		360.1	1.778	85.806
16	-0.0		Mobilogram		BPM +All MS	1278.9602		370.2	1.829	93.253
17	-0.0		Mobilogram		BPM +All MS	1337.0021		380.0	1.878	100.041
18	-0.0		Mobilogram		BPM +All MS	1395.0442		389.6	1.926	111.028
19	-0.0		Mobilogram		BPM +All MS	1453.0862		399.0	1.974	119.350
20	-0.0		Mobilogram		BPM +All MS	1511.1281		407.9	2.018	165.206
21	-0.0		Mobilogram		BPM +All MS	1569.1708		411.8	2.038	191.021

S13. APFD-TIMS analysis of Jeffamine M-2005, #6

Analysis Info

Analysis	D:\Projekte\APFD_TIMS_2024\DataTimsTOF\tims8919_3.d
Method	APFD_pos_100-2500_custom.m
Sample	Jeffamine
Comment	Jeffamin M-2005, 1uL, APFD 4500V+500V 4l 150C EHC<140mATuneMix TIMS 2.5 mbar, custom 0.80-2.10, ramp 500, accum 100 ms, dutycycle 20%, kalib TIMS

Acquisition Date 22.01.2024 15:19:58

Operator TOF-User

Instrument timsTOF fleX 1859745.20462

Acquisition Parameter

Source Type	ESI	Ion Polarity	Positive	Set Nebulizer	0.0 Bar
Scan Begin	100 m/z	Set Capillary	4500 V	Set Dry Heater	150 °C
Scan End	2500 m/z	Set Multipole RF	400.0 Vpp	Set Dry Gas	4.0 l/min
		Set Collision Cell RF	1200.0 Vpp	ICC active	Off
		IMS Active	On	ICC Target	2000000 cts.
		IMS Collision Cell In	300.0 V		

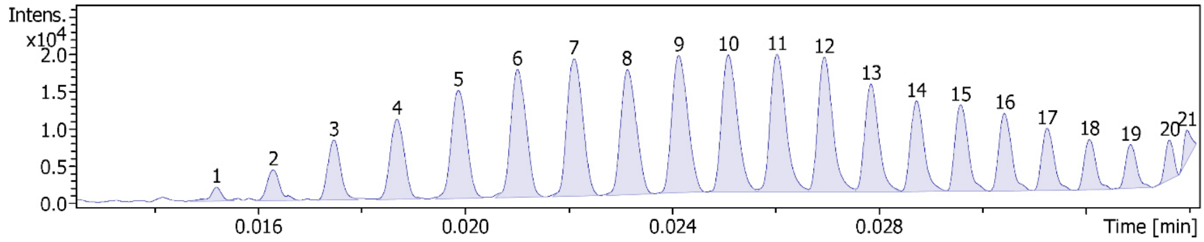

#	RT [min]	Area	Int. Type	I	Trace	Max. m/z	FWHM [min]	CCS [Å²]	Mobility 1/K0	Resolution 1/K0
1	-0.0		Mobilogram		BPM +All MS	408.3323		188.8	0.912	76.981
2	-0.0		Mobilogram		BPM +All MS	466.3737		201.6	0.978	63.293
3	-0.0		Mobilogram		BPM +All MS	524.4150		215.5	1.048	64.828
4	-0.0		Mobilogram		BPM +All MS	582.4566		229.9	1.121	60.992
5	-0.0		Mobilogram		BPM +All MS	640.4984		244.0	1.193	60.639
6	-0.0		Mobilogram		BPM +All MS	698.5404		257.5	1.261	63.502
7	-0.0		Mobilogram		BPM +All MS	756.5822		270.5	1.326	64.519
8	-0.0		Mobilogram		BPM +All MS	814.6238		282.8	1.388	67.699
9	-0.0		Mobilogram		BPM +All MS	872.6661		294.6	1.448	70.712
10	-0.0		Mobilogram		BPM +All MS	930.7078		306.0	1.505	75.598
11	-0.0		Mobilogram		BPM +All MS	988.7503		317.2	1.562	82.097
12	-0.0		Mobilogram		BPM +All MS	1046.7921		328.0	1.617	89.655
13	-0.0		Mobilogram		BPM +All MS	1104.8344		338.7	1.670	96.629
14	-0.0		Mobilogram		BPM +All MS	1162.8766		349.2	1.723	103.342
15	-0.0		Mobilogram		BPM +All MS	1220.9186		359.4	1.775	107.500
16	-0.0		Mobilogram		BPM +All MS	1278.9608		369.5	1.825	114.632
17	-0.0		Mobilogram		BPM +All MS	1337.0026		379.3	1.875	121.398
18	-0.0		Mobilogram		BPM +All MS	1395.0442		389.1	1.924	132.578
19	-0.0		Mobilogram		BPM +All MS	1453.0867		398.6	1.971	140.168
20	-0.0		Mobilogram		BPM +All MS	1511.1281		407.5	2.017	181.428
21	-0.0		Mobilogram		BPM +All MS	1569.1701		411.5	2.037	139.076

S14. ESI mode for *m/z* and TIMS calibration to prepare for next samples. Overall time consumption less than 40 min.

Analysis Info

Analysis	D:\Projekte\APFD_TIMS_2024\DataTimsTOF\tims8920_1.d	Acquisition Date	22.01.2024 15:26:53
Method	APFD_pos_100-2500_custom.m	Operator	TOF-User
Sample	TuneMix	Instrument	timsTOF fleX 1859745.20462
Comment	TuneMix TIMS 2.5 mbar, custom 0.80-2.10, ramp 500, accum 100 ms, dutycycle 20%, kalib TIMS		

Acquisition Parameter

Source Type	ESI	Ion Polarity	Positive	Set Nebulizer	0.5 Bar
Scan Begin	100 m/z	Set Capillary	3800 V	Set Dry Heater	150 °C
Scan End	2500 m/z	Set Multipole RF	400.0 Vpp	Set Dry Gas	4.0 l/min
		Set Collision Cell RF	1200.0 Vpp	ICC active	Off
		IMS Active	On	ICC Target	2000000 cts.
		IMS Collision Cell In	300.0 V		

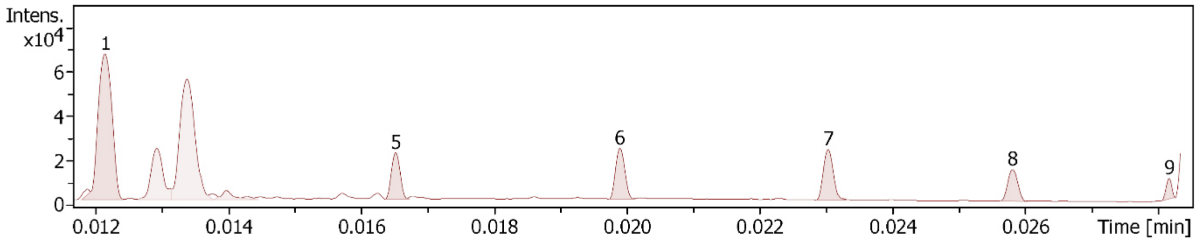

#	RT [min]	Area	Int. Type	I	Trace	Max. m/z	FWHM [min]	CCS [Å²]	Mobility 1/K0	Resolution 1/K0
1	-0.0		Mobilogram		BPM +All MS	322.0480		152.2	0.729	50.141
5	-0.0		Mobilogram		BPM +All MS	622.0284		203.0	0.991	109.610
6	-0.0		Mobilogram		BPM +All MS	922.0080		242.6	1.194	124.686
7	-0.0		Mobilogram		BPM +All MS	1221.9882		279.9	1.382	137.864
8	-0.0		Mobilogram		BPM +All MS	1521.9690			1.548	156.876
9	-0.0		Mobilogram		BPM +All MS	1821.9510		341.0	1.690	281.736

Table S2. CCS determination of $[M+H]^+$ ions of Jeffamine M-2005 by APFD-TIMS-MS.

			CCS [Å2]						CCS [Å2]	
Entry No.	m/z (calc.)	Formula	Run #1	Run #2	Run #3	Run #4	Run #5	Run #6	Average	Stand. Dev. [%]
1	408.3320	C21H46NO6		191.5	191.2	188.9	188.8	188.8	189.8	0.7
2	466.3738	C24H52NO7	204.7	204.0	203.5	201.6	201.6	201.6	202.8	0.6
3	524.4157	C27H58NO8	218.4	217.7	217.3	215.5	215.6	215.5	216.7	0.5
4	582.4576	C30H64NO9	232.5	231.7	231.3	229.9	229.9	229.9	230.9	0.4
5	640.4994	C33H70NO10	246.2	245.3	245.0	244.0	244.1	244.0	244.8	0.3
6	698.5413	C36H76NO11	259.4	258.4	258.0	257.6	257.7	257.5	258.1	0.3
7	756.5832	C39H82NO12	271.9	270.8	270.5	270.5	270.7	270.5	270.8	0.2
8	814.6250	C42H88NO13	283.8	282.6	282.3	282.8	283.1	282.8	282.9	0.2
9	872.6669	C45H94NO14	295.2	293.9	293.7	294.6	295.0	294.6	294.5	0.2
10	930.7087	C48H100NO15	306.2	304.8	304.5	306.2	306.4	306.0	305.7	0.2
11	988.7506	C51H106NO16	317.0	315.5	315.1	317.3	317.7	317.2	316.6	0.3
12	1046.7925	C54H112NO17	327.4	325.9	325.5	328.4	328.6	328.0	327.3	0.4
13	1104.8343	C57H118NO18	337.6	336.1	335.6	339.1	339.3	338.7	337.7	0.4
14	1162.8762	C60H124NO19	347.6	346.0	345.5	349.5	349.8	349.2	347.9	0.5
15	1220.9181	C63H130NO20	357.3	355.7	355.2	359.8	360.1	359.4	357.9	0.5
16	1278.9599	C66H136NO21	366.8	365.2	364.7	369.9	370.2	369.5	367.7	0.6
17	1337.0018	C69H142NO22	376.1	374.4	373.9	379.7	380.0	379.3	377.2	0.7
18	1395.0437	C72H148NO23	385.2	383.3	382.7	389.4	389.6	389.1	386.6	0.8
19	1453.0855	C75H154NO24	392.8		388.7	398.8	399.0	398.6	395.6	1.0
20	1511.1274	C78H160NO25	395.7			407.8	407.9	407.5	404.7	1.3
21	1569.1693	C81H166NO26				411.9	411.8	411.5	411.7	0.0

Table S3 and Fig. S15. Determination of the limits of detection of fluoranthene and benzo[a]pyrene molecular ions by APFD-TIMS-MS. The intensities of the $M^{+\bullet}$ ion peaks were determined three times per sample load and averages of the three runs, I_{avg}, were taken as a measure of intensity. An intensity of a few hundred counts was assumed to represent a useful measure for a still useable spectrum. Thus, the LOD was determined as ≈ 100 pg for fluoranthene and as < 1 pg for benzo[a]pyrene.

Sample Load [pg]	F 1	F1	F3	Flouranthene I_{avg} [Counts]
1000	3140	439	606	1395
100	185	90	217	164
10				
1				

Sample Load [pg]	B1	B3	B3	Benzo[a]pyrene I_{avg} [Counts]
1000				
100	8896	11338	17395	12543
10	2813	2916	1113	2281
1	619	346	645	537

S15.

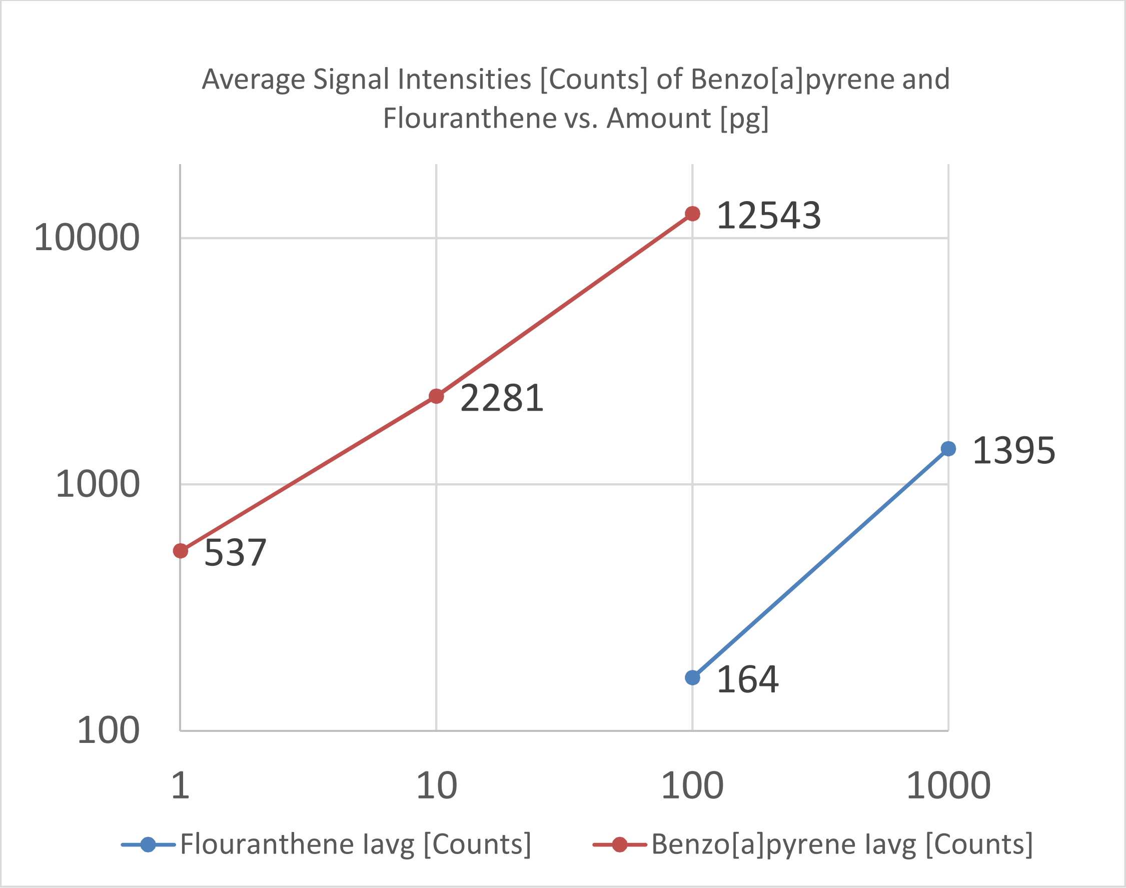

Supplement: Supplementary file 1 — Supplementary file1 (PDF 7722 KB) [file 216_2024_5282_MOESM1_ESM.pdf]
